# Salusin-β, a TOR2A gene product, promotes proliferation, migration, fibrosis, and calcification of smooth muscle cells and accelerates the imbalance of vasomotor function and vascular remodeling in monocrotaline-induced pulmonary hypertensive rats

**DOI:** 10.3389/fphar.2022.928834

**Published:** 2022-09-30

**Authors:** Xingxing Wang, Aidong Chen, Ruihua Hu, Feng Zhang, Shuxin Liang, Changlei Bao, Xuanxuan Liu, Haiyang Tang, Ying Han

**Affiliations:** ^1^ Key Laboratory of Targeted Intervention of Cardiovascular Disease, Collaborative Innovation Center for Cardiovascular Disease Translational Medicine, and Department of Physiology, Nanjing Medical University, Nanjing, Jiangsu, China; ^2^ State Key Laboratory of Respiratory Disease, National Clinical Research Center for Respiratory Disease, Guangzhou Institute of Respiratory Health, The First Affiliated Hospital of Guangzhou Medical University, Guangzhou, Guangdong, China; ^3^ College of Veterinary Medicine, Northwest A&F University, Yangling, China; ^4^ Department of Physiology, Kangda College of Nanjing Medical University, Lianyungang, Jiangsu, China

**Keywords:** pulmonary artrial smooth muscle cells, pulmonary hypertension, vasomotor function, reactive oxygen species, vascular remodeling

## Abstract

**Purpose:** The hyper-proliferation, promoted migration, fibrosis, and calcification of pulmonary arterial smooth muscle cells (PASMCs) play critical roles in pulmonary artery (PA) continuous contraction and vascular remodeling, leading to elevated pulmonary arterial resistance and pulmonary hypertension (PH). In this study, we sought to ascertain the effects of a TOR2A gene product, salusin-β, on PASMCs’ proliferation, migration, fibrosis, calcification, and the imbalance of vasomotor function as well as pulmonary vascular remodeling in monocrotaline (MCT)-induced PH rats and their underlying mechanisms.

**Methods:** Knockdown or overexpression of salusin-β in rats or PASMCs was performed through tail vein injection or cell transfection of virus. The right ventricular systolic pressure (RVSP) of the rat was measured by right ventricle catheterization. Sodium nitroprusside (SNP) or acetylcholine (ACh)-induced dose-dependent relaxation was used to evaluate the vasodilatation function. Primary PASMCs were isolated from the PAs of control and PH rats.

**Results:** The salusin-β protein expressions were significantly increased in PAs and PASMCs isolated from PH rats compared with control rats. Knockdown of salusin-β in rats decreased high K^+^ solution-induced contraction, RVSP and RV hypertrophy index, improved SNP or ACh-induced vascular relaxation of PAs, and relieved vascular remodeling and calcification of PAs from PH rats. Silencing salusin-β in PASMCs isolated from PH rats alleviated the proliferation, migration, fibrosis, and calcification, as well as the NAD(P)H oxidase activity and reactive oxygen species (ROS) level. Overexpression of salusin-β exerted the opposite effects on vasomotor function and vascular remodeling, and PASMCs proliferation, migration, fibrosis and calcification.

**Conclusion:** Increased salusin-β activity in PAs from PH rats contributes to PASMCs proliferation, migration, fibrosis, and calcification, leading to the imbalance of vascular contraction and relaxation and vascular remodeling through stimulating the production of NAD(P)H oxidase derived ROS.

## Introduction

Pulmonary hypertension (PH) is characterized by persistent increases in mean pulmonary arterial pressure (mPAP) and right ventricular systolic pressure (RVSP), which eventually lead to right heart failure with shortness of breath and syncope (Southgate et al., 2020). In the past decade, the death rate of PH has remained at a high level, increasing by 2.5% per year for women and 0.9% for men (Benza et al., 2012). Attenuated relaxation and continuous contraction of pulmonary arteries (PAs), sustained media hyperplasia and fibrosis, and increased wall stiffness lead to the decrease of lumen diameter and even occlusion of PAs, resulting in increases in pulmonary arterial resistance (PAR) and mPAP in PH (Pan et al., 2017; Zabini et al., 2018; Sommer et al., 2021). Pulmonary arterial smooth muscle cells (PASMCs) isolated from patients or animal models with PH exhibit a phenotypic switch from a differentiated state to a dedifferentiated/proliferative state. The structural and functional properties changes of PASMCs due to phenotypic switch are major causes for continuous contraction, increased media thickness (Murray et al., 1990; Brozovich et al., 2016), and migration, fibrosis, and calcification, which vitally decrease vessel elasticity and increase the stiffness of PAs in PH (Humbert et al., 2019; Sommer et al., 2021). However, at present, clinical therapy for PH mainly relies on vasodilators or anticoagulants. Established pharmacological therapies for PH rarely prevent or reverse pulmonary arterial wall thickening, stiffening, and hypercontractility (Gerthoffer, 2020). Therefore, it is important to determine the pathological mechanism of PH during continuous contraction and vascular remodeling for clinical therapy.

Through alternative splicing and RNA rearrangement, the torsin family two member A gene (TOR2A) productions include salusin-*α* and salusin-β, which both can be found in human plasma and urine (Suzuki et al., 2007). They are a class of vascular active peptides, and compared to salusin-α, salusin-β is more related with cardiovascular diseases and abundantly expressed in many organs and tissues including heart, brain, and kidneys, especially in the blood vessels (Suzuki et al., 2007). Recent studies have identified that salusin-β contributes to various diseases including atherosclerosis, hypertension, diabetes and metabolic syndrome (Watanabe et al., 2008). Salusin-β has also been reported to be involved in the regulation of proliferation, migration, fibrosis, and calcification of normal vascular smooth muscle cells (VSMCs) (Sun et al., 2015; Sun et al., 2019). Increased salusin-β expression contributed to vascular inflammation associated with pulmonary arterial hypertension in rats (Xu et al., 2016). However, whether salusin-β is associated with increased vascular tension and regulating the proliferation, migration, fibrosis, and calcification of PASMCs in PH, and its down-steam mechanisms remain unclear.

Therefore using monocrotaline (MCT)-induced PH rat model and derived PASMCs with salusin-β knockdown or overexpression, we evaluated the roles of salusin-β in regulating the proliferation, migration, fibrosis, and calcification of PASMCs, and the roles of it in regulating the imbalance of vasomotor function, vascular remodeling, and the development and progression of pulmonary hypertension, as well as its down-steam mechanisms.

## Materials and methods

### Animals

Male Sprague-Dawley rats were fed in the animal facility, which has controlled temperature and humidity in an automatic 12 h light/dark cycle. Rats were provided with standard chow and tap water *ad libitum*. Animal experiments were carried out according to the procedures approved by the Nanjing Medical University Experimental Animal Care committee and complied with the Guide for the Care and Use of Laboratory Animals published by the US National Institutes of Health (NIH publication, 8th edition, 2011).

### Model of pulmonary hypertension

A single subcutaneous injection of MCT (60 mg/kg) was administered to male rats (200–250 g) to induce PH. Control rats were injected with vehicle (saline). Three weeks after MCT injection, animals were assessed for right ventricular pressure, vascular remodeling, and right ventricular hypertrophy (RVH).

### Knockdown or overexpression of salusin-β in rats or pulmonary arterial smooth muscle cells

PH and control rats were injected with recombinant adeno-associated virus serotype 9 (AAV9) carrying the rat salusin-β short hairpin small–interfering RNA (shRNA, 3.69 × 10^12^ vg/ml, Shanghai Genechem Co., China) and scrambled *via* tail vein to knockdown salusin-β as previously reported (Ren et al., 2017). The AAV9 vectors encoding salusin-β (2.13 × 10^12^ vg/ml, constructed by Shanghai Genechem Co., China) were injected to overexpress the salusin-β gene in rats. The empty vectors were used as the negative control. To determine the validity of AAV9, after 2 weeks, expression level of salusin-β in the PAs of rats was measured using western blot. Knockdown or overexpression of salusin-β in PASMCs was performed by adenovirus infection (100MOI).

### Hemodynamic measurements and evaluation of right ventricular hypertrophy

The rats were weighed and anaesthetized with an intraperitoneal injection of urethane (800 mg/kg). RVP was measured by a catheter connected with a pressure transducer and positioned in the right ventricle (RV) from the right jugular vein as previously described (Tang et al., 2016). The signal from the pressure transducer was recorded using a four-channel bridge amplifier (QUAD bridge, ADInstruments, Australia) and the PoweLab data analysis and processing system (8SP type, ADInstruments, Australia), and the right ventricular systolic pressure (RVSP) was calculated. After measuring the RVSP, the lung and heart of a rat were isolated, the RV, left ventricle (LV), and interventricular septum (S) were separated and weighted. Right ventricular hypertrophy (RVH) index was defined as the ratio of RV weight to the LV plus S weight [(RV/LV+S)].

### Histopathology assessment

Lungs were isolated from rats, fixed in 4% paraformaldehyde, embedded in paraffin, sectioned at 4 μm thick and mounted onto slides. Every slide from different groups was made at same place of lung of rats. Subsequently, the slides were stained with hematoxylin and eosin (H&E), Masson, or von Kossa, and were observed under a light microscope form or photometric assay for PAs wall thickness, lumen diameter, vascular fibrosis, or vascular calcium deposition of PAs, respectively. The PAs wall thickness, lumen diameter, and collagen volume fraction (%) were measured by Image-Pro Plus 6.0 software. The statistics of collagen volume fraction (%), which is equal to the ratio of blue collagen deposition area/total cross-sectional area of blood vessels (%), were used to show the vascular fibrosis quantitatively. The statistics of the amount of calcificated distal PAs/the total amount of distal PAs (%) in one slide were used to show the ratio of calcificated PAs.

### Isometric tension measurements

The tertiary-branch PAs rings (2 mm long) were isolated from rats and were superfused in Krebs-Henseleit solution [the composition was described in a previous report (Zhang et al., 2019)]. Then the PA ring was mounted to the jaw in a four-chambered myograph (620M, DMT, Denmark). In one rat, only one PA ring was used to test the tension response to one drug or one treatment factor. The resting tension was set at 0.1 g. After equilibration, arterial rings were stimulated with the high K^+^ solution [the composition was described as previous reported (Zhang et al., 2019)] to evaluate contractile function. Sodium nitroprusside (SNP) is a nitric oxide (NO) donor which can cause the relaxation of vascular smooth muscle directly. SNP administration to PAs in a dose-dependent manner (10^−9^∼10^−4^ mol/L) after the prostaglandin F2α (PGF 2α) (1–5 μmol/L) induced pre-contraction was performed to evaluate PASMCs relaxing function. Acetylcholine (ACh) stimulates the release of NO in vascular endothelial cells, causing endothelium-dependent relaxation. ACh (10^−9^∼10^−4^ mol/L) administration in a dose-dependent manner after PGF 2α was also performed to evaluate vascular endothelium-dependent relaxation. The degree of relaxation is shown as a percentage of PGF 2α-induced contraction.

### Culture of primary pulmonary arterial smooth muscle cells

PASMCs were isolated from the media of the PAs of control and PH rats, as previously reported (Sun et al., 2016b). After the perivascular adipose tissues and adventitia of the PAs were stripped off with forceps, the endothelium was carefully removed using a cotton swab. The tissues were minced and incubated with type I collagenase for digestion until the shape of the tissues disappeared and then centrifuged to isolate the cells. The isolated primary PASMCs were cultured in DMEM containing 15% fetal bovine serum (FBS), penicillin (100 IU/ml), and streptomycin (10 mg/ml) (Gibco, Grand Island, NY, United States) at 37°C in an incubator with 5% CO_2_ for 5 days. The PASMCs were identified by their substantial smooth muscle-specific α-actin (α-SMA) expression (a marker of VSMCs) without detectable vimentin (a marker of fibroblasts) and platelet endothelial cell adhesion molecule (PECAM)-1 (a marker of endothelial cells) expressions. The overall cell experiments were performed with the second to fifth passage of PASMCs. The same cell passage for each experiment was chosen individually.

### CCK8 test

The proliferation of PASMCs was determined using the CCK8 test. First, PASMCs were seeded in the 96-well plate and the absorbance value was detected with a microplate reader at a wavelength of 450 nm. PASMCs were then subjected to virus infection for 24 h and 48 h. The absorbance values were again measured.

### Wound-healing assay

Migration of PASMCs was assessed using a wound-healing assay as previously reported (Sun et al., 2016b). Briefly, PASMCs were plated into a 6-well plate and cultured to reach confluence. Then, cells were scratched to form a gap using a standard 200 µl pipette tip and were washed with PBS to remove the detached cells. Subsequently, the cells were incubated for 36 h with fresh medium and were observed at 0 and 36 h under an inverted microscope (Axio Vert. A1, Zeiss, Oberkochen, Germany).

### Boyden chamber assay

PASMCs were seeded into FBS-free medium in the upper chamber of a 24-well transwell with an 8 µm pore size (Millipore, United States). The lower chamber was added with a complete medium containing 15% FBS. After being incubated for 24 h, cells on the upper surface of each filter were removed with a cotton swab and migrated cells on the lower surface were stained with crystal violet. Stained cells were counted in five random fields.

### Western blot analysis

The salusin-β protein expression in PAs was detected by western blot as described in our previous reports (Wu et al., 2020). First, lysis buffer was used to extract protein samples and the BCA protein assay kit (BCA, Pierce, United States) was used to detect the protein concentration. Equal amounts of protein samples were added to an SDS-PAGE gel for electrophoresis and then transferred to a polyvinylidene fluoride (PVDF) membrane. The membranes were blocked in 5% skim milk at room temperature for 2 h and were incubated overnight at 4°C with anti-salusin-β IgG (1:1000, Clound-Clone Corp, United States), proliferating cell nuclear antigen (PCNA) antibody (1:2000, Abcam, United States), β-actin antibody (1:5000, Abways Technology Inc., Shanghai, China) or GAPDH antibody (1:10000, Proteintech, China) followed by incubation with horseradish peroxidase-conjugated goat anti-rabbit IgG (1:5000, Immunology Consultants Lab, Portland, OR, United States). Bands were detected with an enhanced chemiluminescence ECL system (Pierce Chemical, Rockford, IL, United States). The expression levels of salusin-β or PCNA proteins were normalized to those of GAPDH or β-actin protein, respectively.

### Measurement of Collagen I, Collagen III, connective tissue growth factor, and tansforming growth factor-β1 mRNA level

The levels of collagen I, collagen III, connective tissue growth factor (CTGF), and tansforming growth factor-β1 (TGF-β1) in PASMCs were used to evaluate the degree of fibrosis as previous report (Sun et al., 2015) and were measured using qPCR. Briefly, RNA was extracted with RNA-easy Isolation Reagent (Nuo Wei Zan, Biotechnology, China) according to the manufacturer’s protocol. PrimeScript^®^ RT reagent kits (Takara, Otsu, Shiga, Japan) and an ABI PRISM 7500 sequence detection PCR system (Applied Biosystems, Foster City, CA, United States) were used for quantitative RT-PCR. Fold-change of RNA was calculated using the 2^−ΔΔCt^, normalized to GAPDH expression. The primer sequences used are shown in Table 1.

**TABLE 1:** Primer pairs used in this study.

Primer sequence (5′–3′)		
Collagen I	Forward	TGG​TGG​TTA​TGA​CTT​TGG​TTA​CGA​T
	Reverse	TGT​GCG​AGC​TGG​GTT​CTT​TCT​A
Collagen III	Forward	CTC​CTG​CAG​GCT​AGA​GAA​GC
	Reverse	GAT​GCA​CTT​TTT​GCC​CTT​CTT
CTGF	Forward	CAG​GGA​GTA​AGG​GAC​ACG​A
	Reverse	ACA​GCA​GTT​AGG​AAC​CCA​GAT
TGF-β1	Forward	GCT​CCA​CGG​AGA​AGA​ACT​GCT
	Reverse	CTG​CTC​CAC​CTT​GGG​CTT​GC
GAPDH	Forward	GGAAAGCTGTGGCGTGAT
	Reverse	AAG​GTG​GAA​GAA​TGG​GAG​TT

### Measurement of NAD(P)H oxidase activity and reactive oxygen species levels

To measure NAD(P)H oxidase activity and ROS levels, the enhanced lucigenin-derived chemiluminescence method was utilized, as we previously reported (Sun et al., 2016b; Lu et al., 2017). The dark-adapted lucigenin was added to PASMCs protein supernatants and reacted with the superoxide anions to induce the photon emission, which can be detected using a luminometer (Model 20/20n, Turner, CA, United States) for 1 time every 1 min until 10 times to represent the ROS level. To detect NAD(P)H oxidase activity, PASMCs protein supernatants were incubated with NAD(P)H (100 µM) to generate new superoxide anions before lucigenin application. The mean light unit (MLU) per minute per milligram of protein represents the values of ROS level and NAD(P)H oxidase activity.

### Detection of alkaline phosphatase activity and calcium content

ALP activity was measured using an ALP assay kit (Nanjing Jiancheng Bioengineering Institute, Nanjing, China) according to the manufacturer’s instructions as our previous reports (Zhou et al., 2013; Sun et al., 2019). The absorbance was determined at 520 nm, and the results were normalized to the protein content of each sample. Calcium content was measured using the o-cresolphthalein colorimetric method as previously described (Ma et al., 2016). The calcium levels in PASMCs samples were normalized to the protein content, as determined by the BCA method (Zhu et al., 2018).

### Statistical analysis

Data are expressed as mean ± S.E. SPSS analysis software was used to perform statistical analysis. A comparison of means between two groups was performed using an unpaired Student’s *t*-test. One-way or two-way ANOVA, followed by the Bonferroni test for post-hoc analysis, was performed when multiple comparisons were made. Statistical significance was set at *p* < 0.05.

## Results

### The salusin-β expressions in pulmonary arteries or pulmonary arterial smooth muscle cells in monocrotaline-induced PH and control rats

**FIGURE 1:**
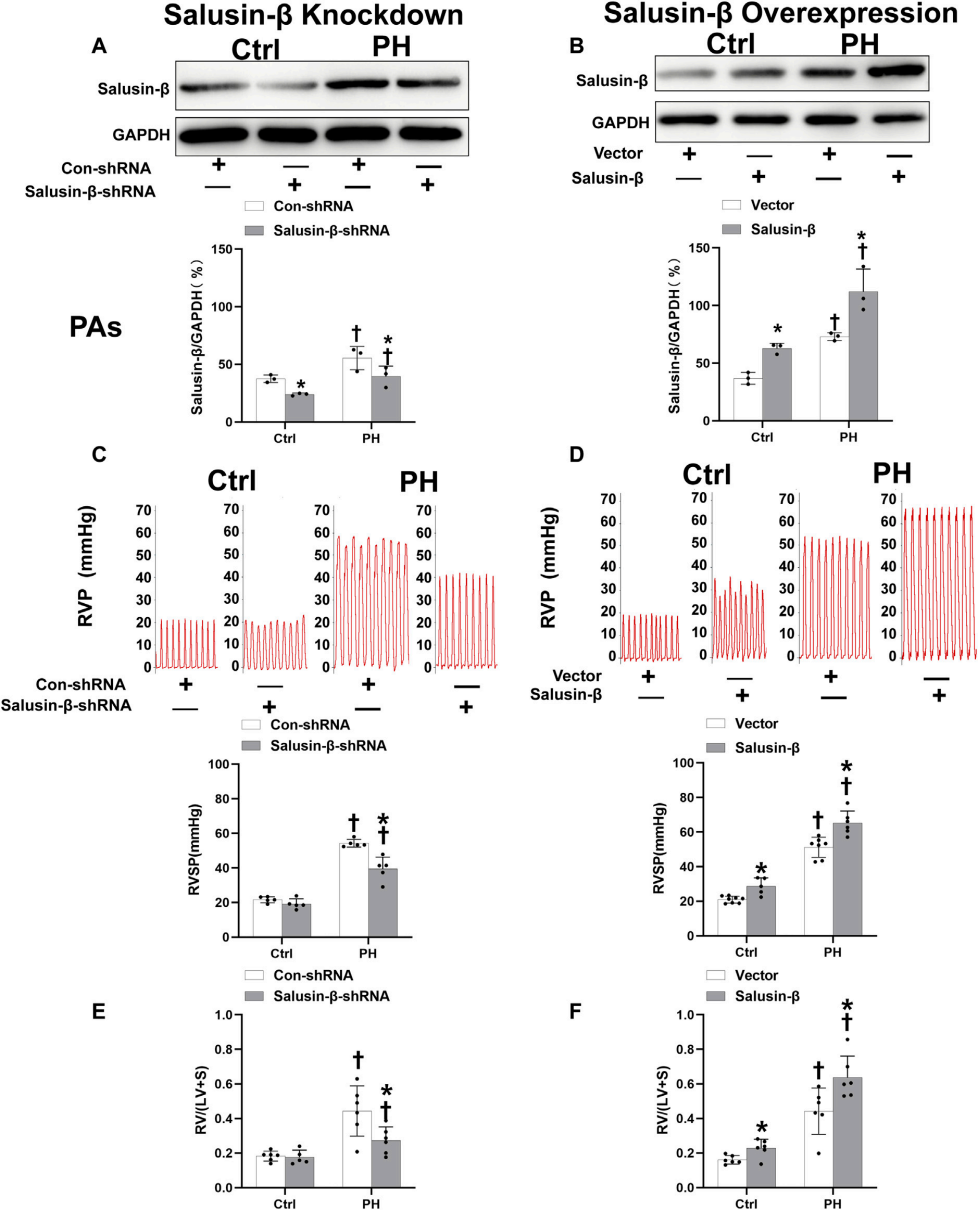
Effects of salusin-β knockdown on salusin-β protein expressions in PAs **(A)**, right ventricular pressure (RVP) and right ventricular systolic pressure (RVSP) **(C)**, and right ventricular hypertrophy index (RVHI) [RV/(LV + S)] **(E)**; as well as the effects of overexpression of salusin-β on its protein expressions in PAs **(B)**, RVP and RVSP **(D)**, and RVHI **(F)** in control and MCT-induced PH rats. Values are mean ± SE. *p < 0.05 compared with Con-shRNA or vector. ^†^ *p* < 0.05 compared with control (Ctrl) rats. *n* = 5–8 for each group.

The protein expressions of salusin-β in both PAs (Figures 1A,B) and PASMCs (Figures 5A,B) of MCT-induced PH rats were higher than that in control rats. Knockdown of salusin-β gene in either rats or PASMCs decreased its expression, and overexpression of salusin-β significantly increased its expression, which confirmed the effectiveness of knockdown or overexpression of salusin-β in rats or PASMCs.

### Effects of salusin-β knockdown or overexpression on RVSP, RVH index, high K^+^ solution-induced contraction and SNP or ACh-induced relaxation of PAs

**FIGURE 2:**
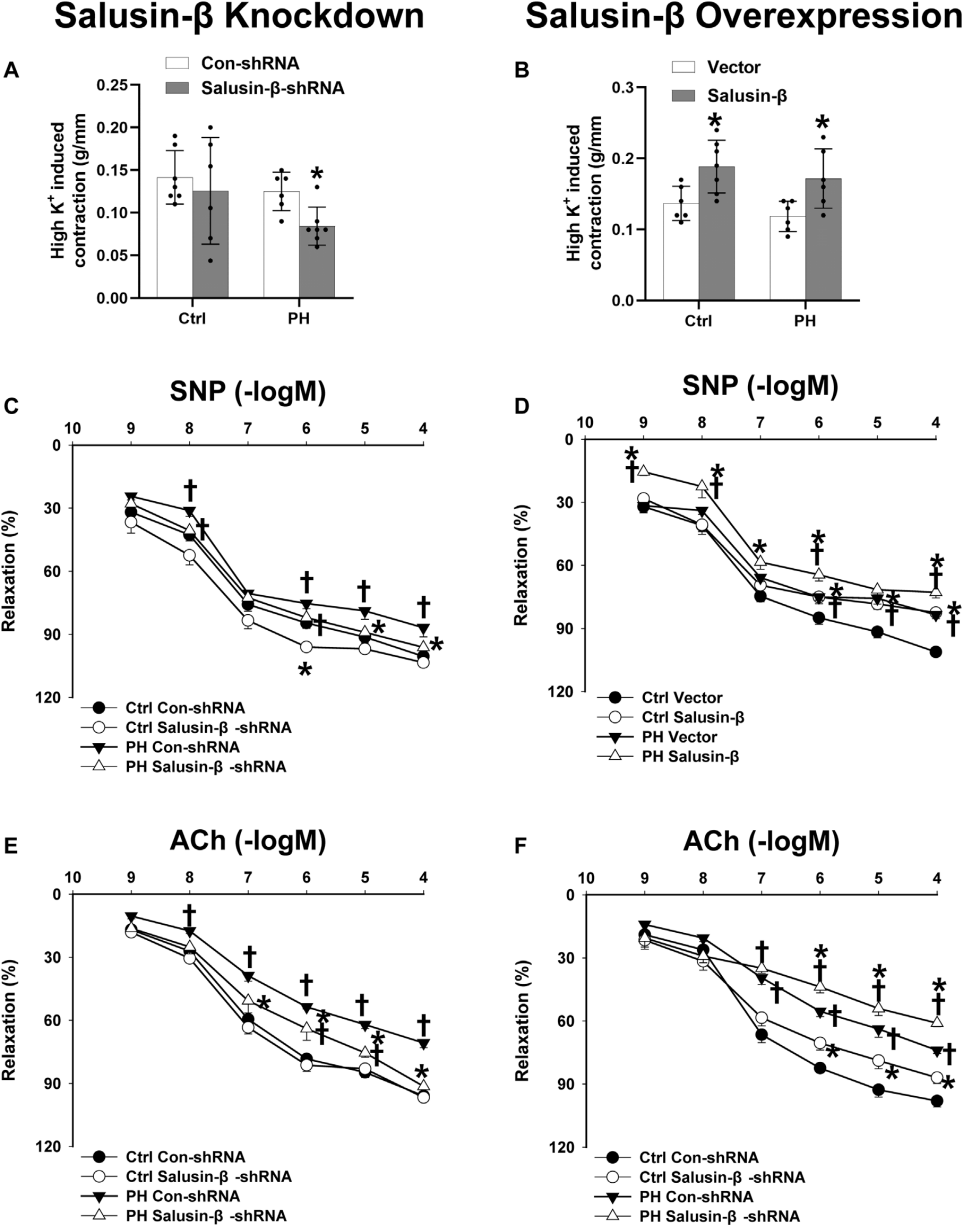
Effects of salusin-β knockdown on high K^+^ solution-induced contraction **(A)**, sodium nitroprusside (SNP)-induced relaxation **(C)**, and acetylcholine (ACh)-induced relaxation of PAs **(E)**; as well as the effects of overexpression of salusin-β on high K+ solution-induced contraction **(B)**, SNP-induced relaxation **(D)**, and ACh-induced relaxation of PAs **(F)** in control and MCT-induced PH rats. Values are mean ± SE. *p < 0.05 compared with Con-shRNA or vector. ^†^ *p* < 0.05 compared with control (Ctrl) rats. *n* = 6–9 for each group.

Compared to control rats, both the RVSP (Figures 1C,D) and RVH index (Figures 1E,F) of MCT-PH rats were significantly higher. There was no significant difference in high K^+^ solution-induced PAs contraction between control and PH rats (Figures 2A,B), whereas both the SNP- and ACh-induced dose-dependent vasodilatation of PAs were significantly attenuated in PH rats **(** Figures 2C–F). Silencing of salusin-β gene in PH rats significantly decreased the RVSP (Figure 1C), RVH index (Figure 1E) and high K^+^ solution-induced PAs contraction (Figure 2A), improved either SNP- (Figure 2C) or ACh-induced endothelium-independent vascular relaxation (Figure 2E), while overexpression of salusin-β worsened the above parameters in PH rats. It was worth noting that although knockdown of salusin-β had no significant roles in control rats, overexpression of salusin-β in control rats elevated the RVSP, RVH index, PAs contraction and attenuated SNP or ACh-induced PA relaxation (Figures 1D,F, 2B,D,F). The pEC50 and Rmax (%) data of SNP or ACh-induced dose-dependent vasodilatation of PAs in control and PH rats with different pretreatments were shown in Table 2, which suggested similar results.

**TABLE 2:** pEC50 and Rmax (%) data of SNP or ACh-induced dose-dependent vasodilatation of PAs in Ctrl and PH rats with different pretreatments.

	Ctrl		PH	
	pEC50	Rmax	pEC50	Rmax
SNP				
Con-shRNA	7.59 ± 0.19	100.59 ± 2.22	7.43 ± 0.07	86.82 ± 4.33^†^
Salusin-β-shRNA	7.63 ± 0.12	103.60 ± 1.68	7.39 ± 0.12	96.17 ± 1.99*
Vector	7.36 ± 0.11	101.14 ± 0.60	7.31 ± 0.07	83.49 ± 1.84^†^
Salusin-β	7.43 ± 0.13	82.44 ± 2.13*	7.51 ± 0.19	73.63 ± 2.94*^†^
ACh				
Con-shRNA	7.17 ± 0.06	95.98 ± 1.49	6.98 ± 0.21	70.75 ± 2.26^†^
Salusin-β-shRNA	7.69 ± 0.15*	96.71 ± 1.21	6.84 ± 0.23^†^	91.38 ± 2.21*
Vector	7.17 ± 0.12	98.03 ± 2.89	6.79 ± 0.29	75.63 ± 0.73^†^
Salusin-β	7.61 ± 0.43	96.01 ± 0.96	6.27 ± 0.28^†^	63.34 ± 0.86*^†^

SNP, sodium nitroprusside; ACh, acetylcholine. Values are mean ± SE. *p < 0.05 vs. Con-shRNA or vector. ^†^ *p* < 0.05 vs. Ctrl. *n* = 6–9 for each group.

### Effects of salusin-β knockdown or overexpression on vascular remodeling of pulmonary arteries

**FIGURE 3:**
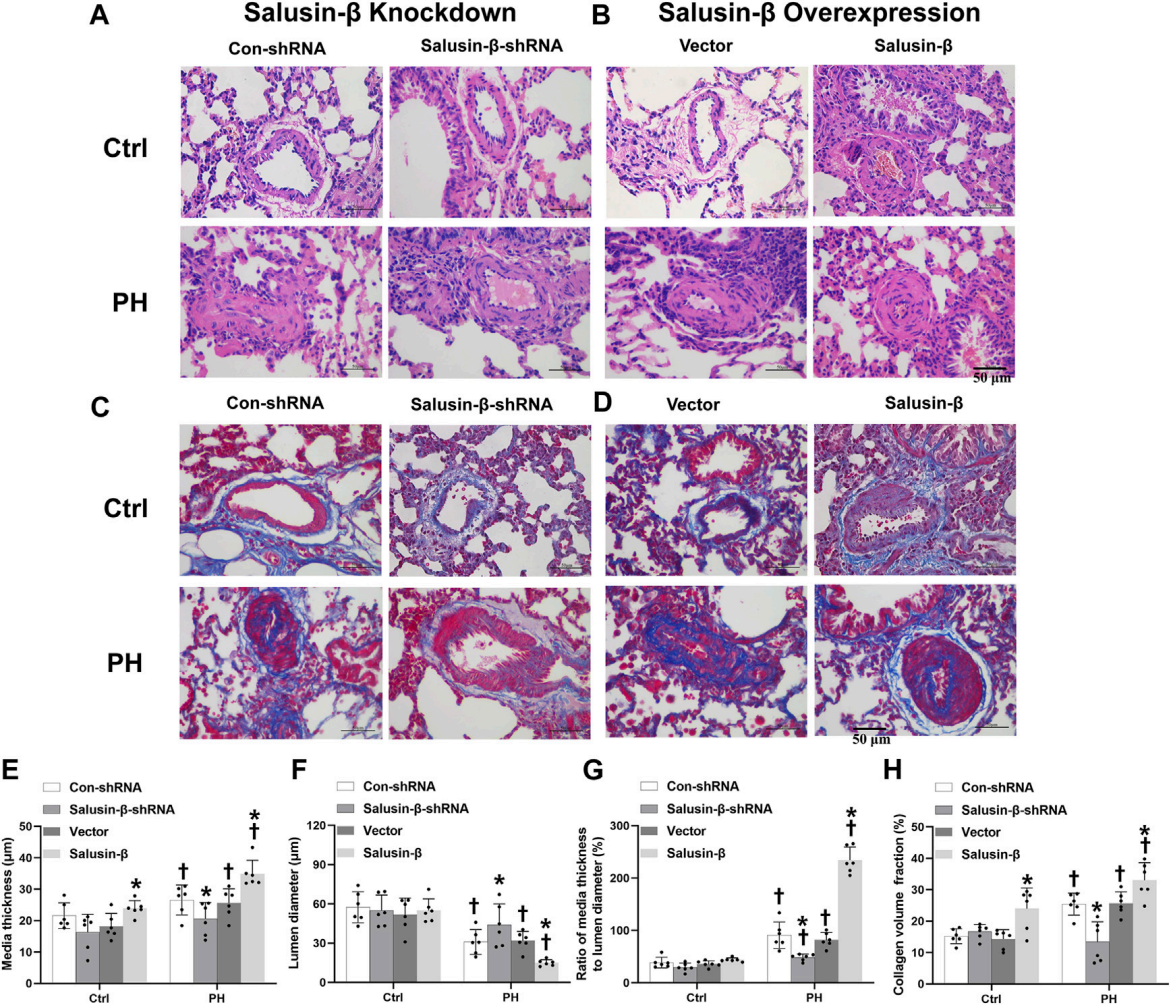
Representative images of lung tissue cross-sections with H and E staining **(A)** and Masson’s staining (Blue indicates collagen deposition) **(C)** showing the effects of salusin-β knockdown on PAs vascular remodeling and fibrosis; lung tissue cross-sections with H and E staining **(B)** and Masson’s staining **(D)** showing the effects of salusin-β overexpression on PAs vascular remodeling and fibrosis. The effects of salusin-β knockdown or overexpression on the media thickness **(E)**, lumen diameter **(F)**, media thickness/lumen diameter **(G)** and collagen volume fraction (%) **(H)** of PAs in control and MCT-induced PH rats. Values are mean ± SE. *p < 0.05 compared with Con-shRNA or vector. ^†^ *p* < 0.05 compared with control (Ctrl) rats. *n* = 6 for each group.

From the representative H&E staining of lung tissues, we observed a significant increase in PA wall thickness and a decrease in lumen diameter, resulting in stenosis or even complete occlusion of PAs in MCT-PH rats compared to control rats (Figure 3A,B). The statistical graph also showed that the lumen diameter of PAs was decreased (Figure 3F), while media thickness (Figure 3E) and media thickness/lumen diameter of PAs (Figure 3G) in PH rats were significantly increased. Additionally, Masson staining of lung tissue images and statistical collagen volume fraction (%) showed severe vascular fibrosis of PAs in PH rats (Figures 3C,D,H). These results suggest the occurrence of vascular remodeling of PAs in PH rats. PAs from PH rats with salusin-β knockdown presented increased lumen diameter, reduced media thickness, with a lower media thickness/lumen diameter ratio, and decreased vascular fibrosis, while salusin-β overexpression caused more severe vascular remodeling in PH rats (Figure 3).

### Effects of salusin-β knockdown or overexpression on vascular calcification of pulmonary arteries

**FIGURE 4:**
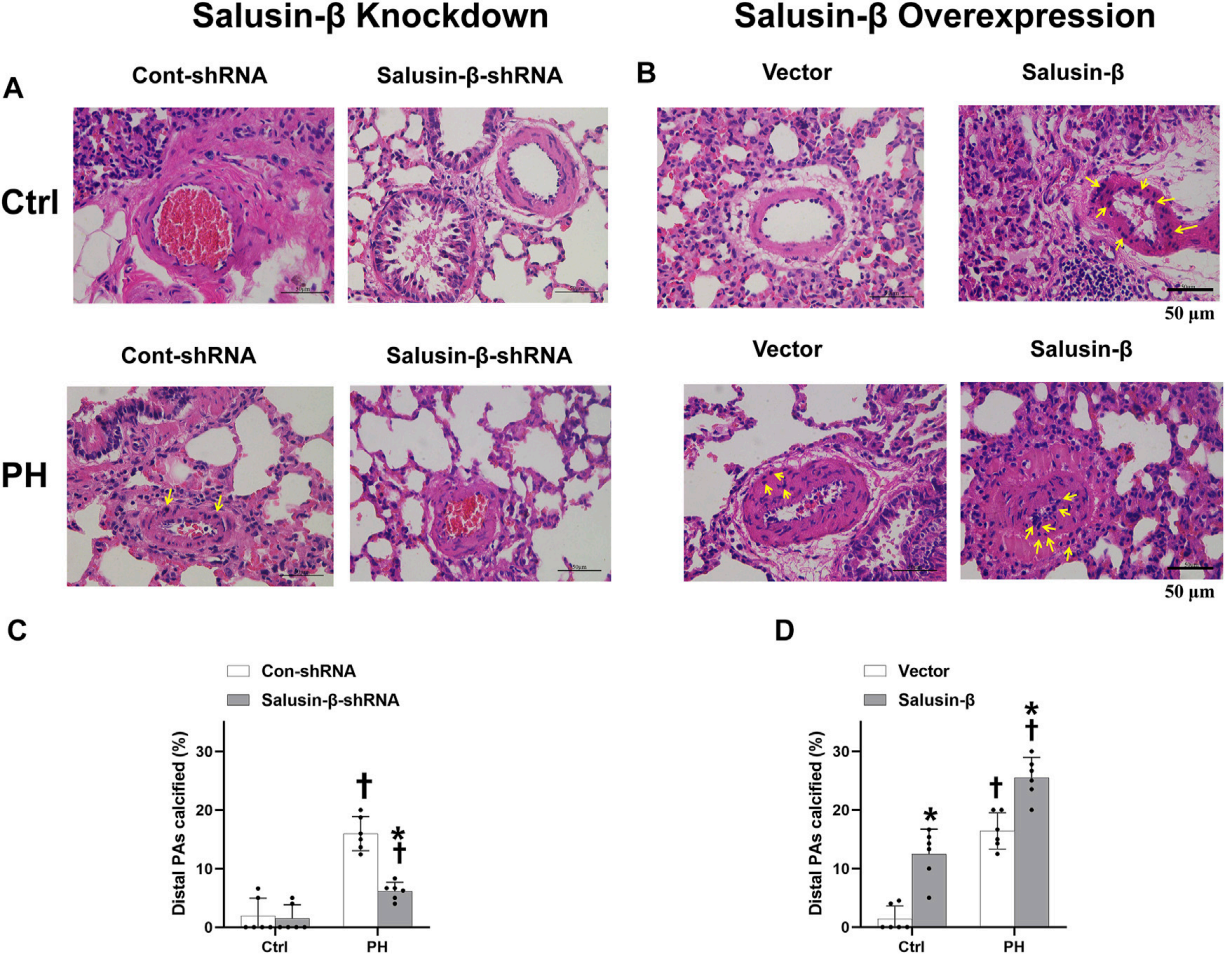
Representative images of lung tissue cross-sections with Von Kossa staining (The calcium deposits are black spots, with blue nuclei and a red background), in which the representative calcium deposits were indicated by yellow arrows **(A)** and statistical percentage of calcified PAs **(C)** showing the effects of salusin-β knockdown on vascular calcification in PAs; images of lung tissue cross-sections with Von Kossa staining **(B)** and percentage of calcified PAs **(D)** showing the effects of salusin-β overexpression on vascular calcification in PAs. Values are mean ± SE. *p < 0.05 compared with Con-shRNA or vector. ^†^ *p* < 0.05 compared with control (Ctrl) rats. *n* = 6 for each group.

The representative Von Kossa staining of lung tissues and statistical data on the percentage of calcified PAs showed that PH rats had a certain degree of vascular calcification in the PAs (Figure 4). Knockdown of salusin-β decreased the degree of calcification and percentage of calcified PAs (Figures 4A,C), whereas the overexpression of salusin-β played the opposite role (Figures 4B,D) in MCT-PH rats. Furthermore, overexpression of salusin-β in control rats induced vascular calcification in the PAs (Figures 4B,D).

### Effects of salusin-β knockdown or overexpression on pulmonary arterial smooth muscle cells proliferation

**FIGURE 5:**
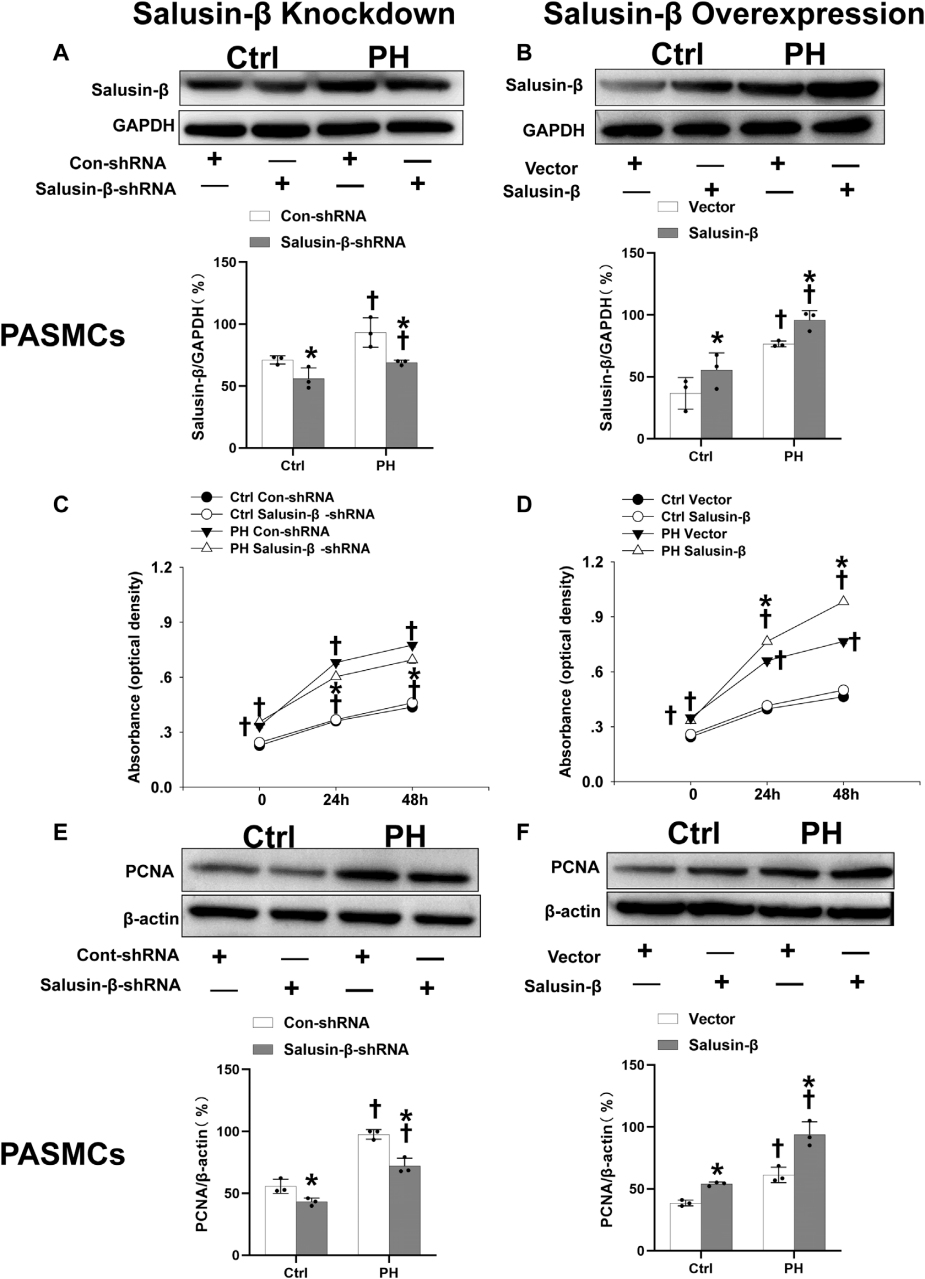
Effects of salusin-β knockdown on salusin-β protein expressions of PASMCs **(A)**, cell viability tested by CCK-8 assay **(C)** and protein expressions of proliferation marker proliferating cell nuclear antigen (PCNA) **(E)**; as well as the effects of overexpression of salusin-β on salusin-β protein expressions **(B)**, cell viability tested by CCK-8 assay **(D)** and proliferation marker PCNA protein expressions **(F)** in PASMCs isolated from control and MCT-induced PH rats. Values are mean ± SE. *p < 0.05 compared with Con-shRNA or vector. ^†^ *p* < 0.05 compared with control (Ctrl) rats. *n* = 3 for A, B, E, and F groups. *n* = 6 for C and D groups.

The results of CCK8 (Figures 5C,D) and elevated PCNA expression (Figures 5E,F) of PASMCs in PH rats showed that PASMC proliferation in PH rats was significantly increased compared to that in control rats. Salusin-β knockdown in PASMCs inhibited proliferation (Figures 5C,E), while overexpression of salusin-β further promoted the proliferation of PASMCs derived from PH rats (Figures 5D,F). In addition, the significant change was not found in silencing salusin-β in control rats, whereas enhanced PASMCs proliferation was observed in control rats with overexpression of salusin-β (Figure 5).

### Effects of salusin-β knockdown or overexpression on pulmonary arterial smooth muscle cells migration

**FIGURE 6:**
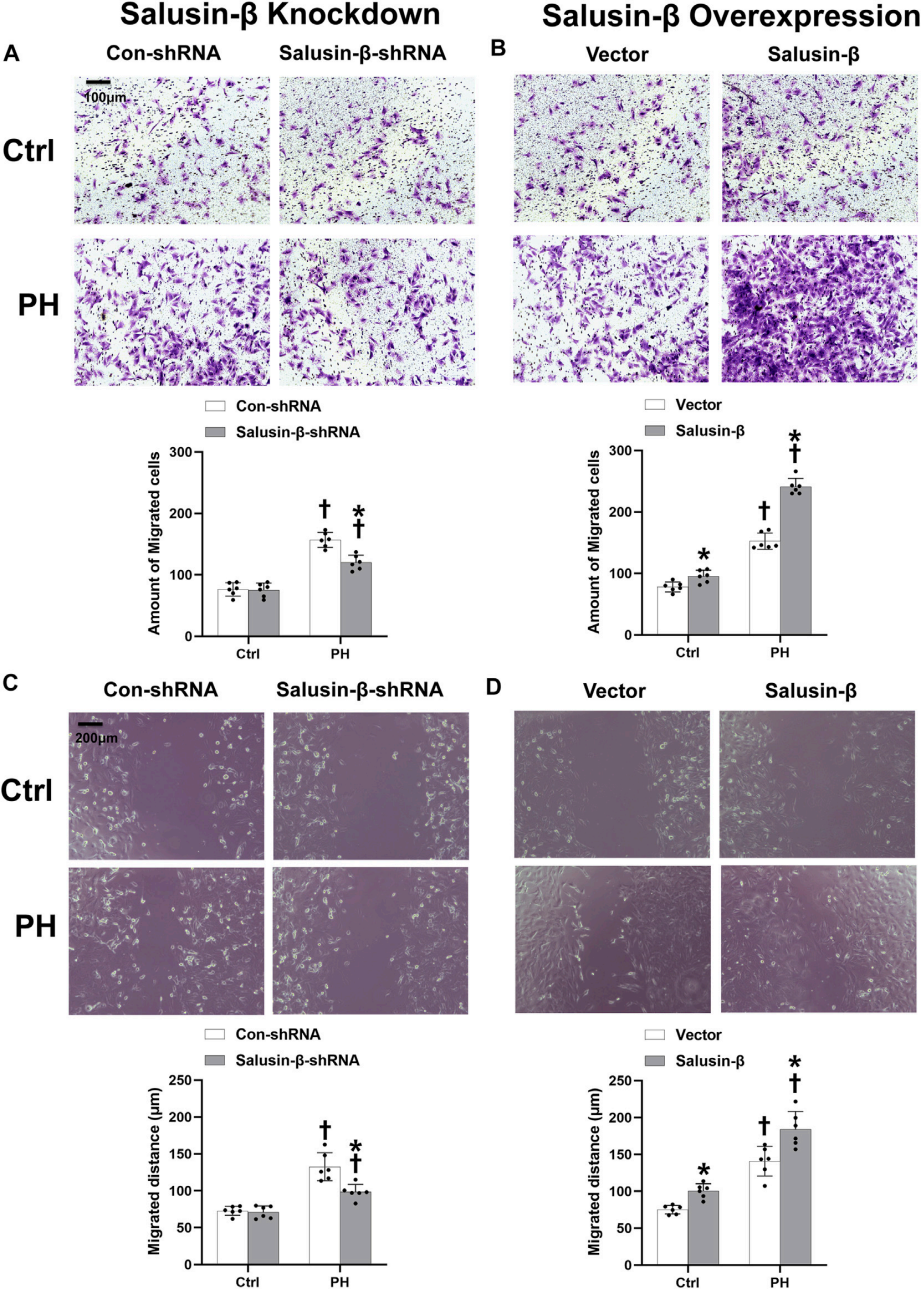
Effects of salusin-β knockdown on PASMCs migration evaluated by the boyden chamber assay **(A)** and a wound healing assay **(C)**; as well as the effects of overexpression of salusin-β on PASMCs migration evaluated by the boyden chamber assay **(B)** and a wound healing assay **(D)** in control and MCT-induced PH rats. Values are mean ± SE. *p < 0.05 compared with Con-shRNA or vector. ^†^ *p* < 0.05 compared with control (Ctrl) rats. *n* = 6 for each group.

Boyden chamber assay (Figures 6A,B) and wound-healing assay results (Figures 6C,D) showed that PASMCs migration in PH rats was enhanced compared to that in control rats. Overexpression of salusin-β promoted the migration of PASMCs derived from both PH rats and control rats (Figures 6B,D), whereas knockdown of salusin-β only reduced the migration of PASMCs in PH rats (Figures 6A,C).

### Effects of salusin-β knockdown or overexpression on pulmonary arterial smooth muscle cells fibrosis

**FIGURE 7:**
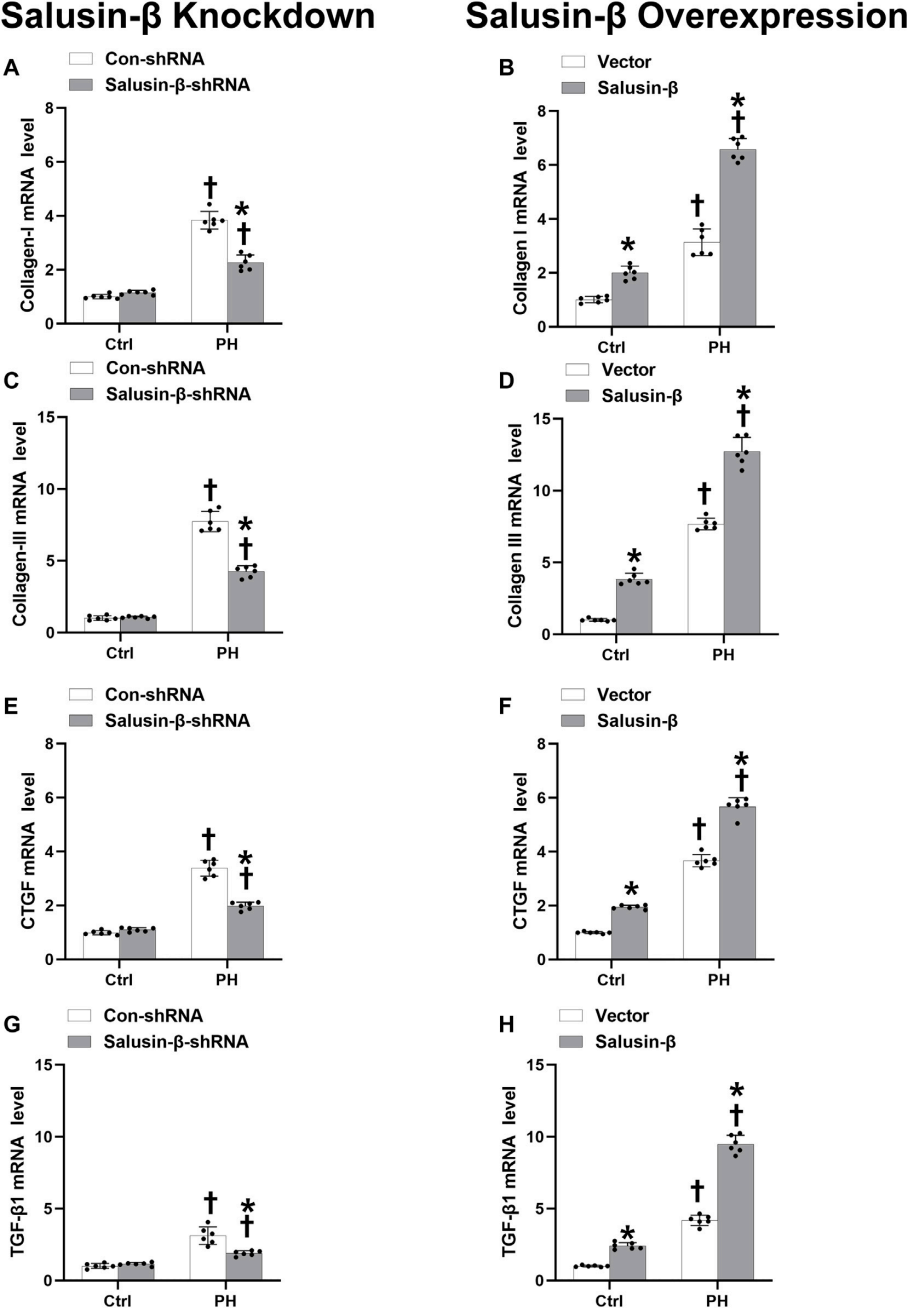
Effects of salusin-β knockdown on mRNA levels of fibrosis indexes of PASMCs: collagen I **(A)**, collagen III **(C)**, connective tissue growth factor (CTGF) **(E)** and transforming growth factor-β1 (TGF-β1) **(G)**; as well as the effects of overexpression of salusin-β on collagen I **(B)**, collagen III **(D)**, CTGF **(F)** and TGF-β1 **(H)** of PASMCs isolated from control and MCT-induced PH rats. Values are mean ± SE. *p < 0.05 compared with Con-shRNA or vector. ^†^ *p* < 0.05 compared with control (Ctrl) rats. *n* = 6 for each group.

The mRNA levels of collagen I (Figures 7A,B), collagen III (Figures 7C,D), CTGF (Figures 7E,F), and TGF-β1 (Figures 7G,H) in the PASMCs of control rats and MCT-PH rats were measured to evaluate the degree of fibrosis. The ΔCT values of each group were shown in Table 3.

**TABLE 3:** The ΔCT values for Collagen-I, Collagen-III, CTGF, and TGF-β1 during mRNA measurement using quantitative RT-PCR.

	Collagen-I		Collagen-III	
	**Ctrl**	**PH**	**Ctrl**	**PH**
Con-shRNA	5.53 ± 0.043	3.59 ± 0.05^†^	7.09 ± 0.09	4.14 ± 0.07^†^
Salusin-β-shRNA	5.33 ± 0.03	4.36 ± 0.06*^†^	6.99 ± 0.10	5.00 ± 0.09*^†^
Vector	5.52 ± 0.06	3.89 ± 0.05^†^	6.97 ± 0.05	4.03 ± 0.04^†^
Salusin-β	4.53 ± 0.08*	2.80 ± 0.04*^†^	5.03 ± 0.06	3.30 ± 0.07*^†^

	**CTGF**		**TGF-β1**	
	**Ctrl**	**PH**	**Ctrl**	**PH**
Con-shRNA	1.19 ± 0.04	−0.56 ± 0.06^†^	2.78 ± 0.09	1.17 ± 0.07^†^
Salusin-β-shRNA	1.05 ± 0.06	0.20 ± 0.02*^†^	2.59 ± 0.12	1.87 ± 0.09*^†^
Vector	1.09 ± 0.02	−0.78 ± 0.02^†^	2.50 ± 0.03	0.44 ± 0.04^†^
Salusin-β	1.03 ± 0.02*	−1.41 ± 0.04*^†^	1.24 ± 0.04*	−0.75 ± 0.03*^†^

Values are mean ± SE. *p < 0.05 vs. Con-shRNA or vector. ^†^ *p* < 0.05 vs. Ctrl. *n* = 6 for each group.

We found that the mRNA levels of these fibrosis indices in PASMCs from MCT-PH rats were higher compared to those in control rats, which suggested that PASMC fibrosis was increased in PH. Knockdown of salusin-β attenuated, while the overexpression of salusin-β enhanced the fibrosis of PASMCs from PH. Similarly, knockdown of salusin-β had no significant roles, while overexpression of salusin-β also elevated the PASMCs fibrosis index of control rats (Figure 7).

### Effects of salusin-β knockdown or overexpression on pulmonary arterial smooth muscle cells calcification and oxidative stress

**FIGURE 8:**
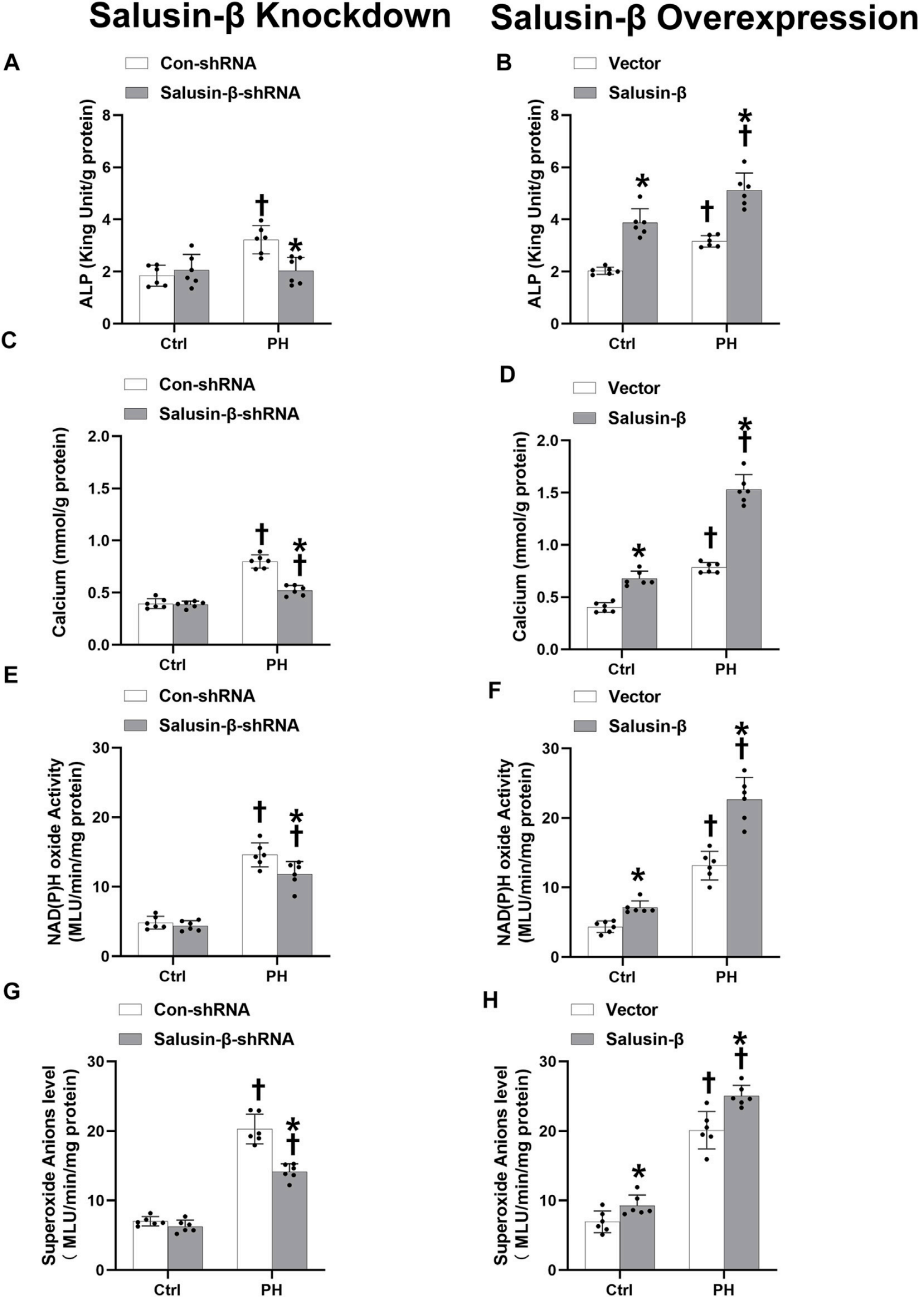
Effect of salusin-β knockdown on ALP activity **(A)**, calcium content **(C)**, NAD(P)H oxidase activity **(E)** and superoxide anions level **(G)**; as well as the effects of overexpression of salusin-β on ALP activity **(B)**, calcium content **(D)**, NAD(P)H oxidase activity **(F)** and superoxide anions level **(H)** of PASMCs isolated from control and MCT-induced PH rats. Values are mean ± SE. *p < 0.05 compared with Con-shRNA or vector. ^†^ *p* < 0.05 compared with control (Ctrl) rats. *n* = 6 for each group.

ALP activity is an indicator of calcification. Compared with PASMCs of control rats, both the ALP activity (Figures 8A,B) and calcium content (Figures 8C,D) in PASMCs of PH rats were higher, which indicated that the PH-PASMC calcification level was elevated. NAD(P)H oxidase activity (Figures 8E,F) and superoxide anions level (Figures 8G,H) in PASMCs of PH rats were also higher than those in PASMCs of control rats, suggesting the occurrence of oxidative stress. Silencing of salusin-β reversed the high ALP activity, calcium content, NAD(P)H oxidase activity, and superoxide anions level in PASMCs derived from PH rats, but had no significant effect on PASMCs from control rats. Overexpression of salusin-β increased the above indices of PASMCs derived from either PH rats or control rats (Figure 8).

## Discussion

The proliferation, migration, fibrosis, and calcification of PASMCs are the major causes of vascular remodeling, which increases the media wall thickness and stiffness, narrows the blood vessel, and ultimately increases PAR and PAP in PH(Tang et al., 2015; Tang et al., 2016; Tanguay et al., 2019; Gerthoffer, 2020). Our previous studies reported that the TOR2A gene product, salusin-β, participates in the regulation of proliferation, migration, fibrosis, and calcification of normal VSMCs(Sun et al., 2015; Sun et al., 2019), as well as the regulation of vasoconstriction and vasodilatation function (Pan et al., 2021; Sun et al., 2021). Knockdown of salusin-β improves vascular relaxation, alleviates vascular remodeling, and decreases vasoconstriction in spontaneously hypertensive rats (Pan et al., 2021; Sun et al., 2021). However, whether salusin-β has similar effects in mediating morphology and function of PASMCs and vascular remodeling, as well as the vasoconstriction and vascular relaxation of PA in PH is still unclear. The present study found that the increased salusin-β activity of PAs in MCT-induced PH rats contributed to the proliferation, migration, fibrosis, and calcification of PASMCs, vascular remodeling, and the imbalance of vascular contraction and relaxation in MCT-induced PH through stimulating the production of NAD(P)H oxidase derived ROS. Reducing endogenous salusin-β levels by salusin-β gene knockdown decreased contractions of PAs, RVSP, and RV hypertrophy, improved the vascular relaxation and prevented the vascular remodeling in PH, while overexpression of salusin-β in rats or PASMCs deteriorates the symptom of PH, which provide a potential therapeutic target for PH.

The TOR2A gene product, salusin-β, is present in human plasma and urine and widely expressed in the lung (Suzuki et al., 2007), and is particularly abundant in vascular endothelial cells and VSMCs (Watanabe et al., 2008). We recently found that salusin-β participates in regulating the vascular constriction and relaxation function in spontaneously hypertensive rats (Pan et al., 2021; Sun et al., 2021). However, whether salusin-β also plays a regulating role in vasomotor function in pulmonary hypertension is still unknown.

The current study found that compared to control rats, the salusin-β protein expressions in both PAs and PASMCs were significantly increased in MCT-PH rats. The high K^+^ solution-induced contraction is usually used to evaluate the contractile ability of vascular smooth muscle; and SNP, an NO donor, which acts on the VSMCs to directly induce vascular smooth muscle relaxation, can be used to evaluate the vasodilator ability of vascular smooth muscle. ACh stimulates endothelial cells to release NO, and NO then causes VSMCs to relax, which is called endothelium-dependent relaxation. We found that compared with control rats, both the SNP- and ACh-induced relaxation of PAs of MCT-PH rats were attenuated, indicating that both the endothelial function and the smooth muscle relaxing ability of PAs were impaired in PH. Tail vein injection of salusin-β shRNA improved either SNP-or ACh-induced vascular relaxation and decreased high K^+^ solution-induced PAs contraction, while overexpression of salusin-β enhanced PAs contraction and worsened vascular relaxation in MCT-PH rats. In addition, although knockdown of salusin-β had no significant roles in control rats, overexpression of salusin-β in control rats similarly elevated PAs contraction and impaired SNP- or ACh-induced PAs relaxation. These results suggested that salusin-β has ability to enhance contraction, and impair endothelial function and PASMCs relaxation, and through that, it consequently increases PAR and PAP.

Furthermore, it is known that high K^+^ solution-elicited contraction is mainly related either to smooth muscle mass or to the function of voltage-dependent calcium channels in pulmonary vascular smooth muscle (Karaki et al., 1986; Ishida et al., 2017). SNP-mediated vasodilatation is related to signaling dependent on soluble guanylate cyclase (GC)-cGMP-production-protein kinase G (PKG) signaling (Bilodeau-Goeseels, 2007). We speculated that salusin-β enhanced contraction might through increasing smooth muscle mass or enhancing the function of voltage-dependent calcium channels of PASMCs, and impaired PASMCs relaxation might through inhibiting GC-cGMP-PKG signaling, which would be studied in the future.

We further found that the RVSP and RVH index in MCT-PH rats were much higher than those of control rats, which were the subsequent results of the high PA pressure. Knockdown of salusin-β decreased RVSP and RVH index in PH rats, while overexpression of salusin-β not only increased RVSP and RVH index in MCT-PH rats, but also increased these parameters in control rats, which further suggested that salusin-β contributes to the pathogenesis and development of PH by influencing the vascular contraction and relaxation function of PAs.

Studies have reported that PASMCs undergo phenotypic changes under pathological conditions in PH, resulting in enhanced proliferation and migration and vascular remodeling (Tajsic and Morrell, 2011; Morris et al., 2019). Excessive hyperplasia and hypertrophy of PASMCs have become the most important pathological basis for pulmonary artery stenosis and increased PAR (You et al., 2018). Fibrosis and calcification of smooth muscles, which increases the stiffness of PAs, are also important causes for the vascular remodeling of pulmonary hypertension (Chan and Loscalzo, 2008; Ruffenach et al., 2016). Vascular fibrosis includes the deposition of extracellular matrix in the vascular wall, especially the accumulation of collagen and fibronectin. Excessive synthesis and deposition of collagen in the vascular wall increases vascular hardness (Lan et al., 2013; Thenappan et al., 2018). Since the 1960s, researchers have found that similar to other vascular lesions involving arterial thickening (e.g., atherosclerosis), vascular calcification is also ubiquitous and is associated with an increased risk of death in pulmonary hypertension (Ruffenach et al., 2016; Tanguay et al., 2019). Studies have suggested that salusin-β contributes to the progression of cardiovascular diseases such as atherosclerosis and hypertension, by regulating the proliferation, migration, fibrosis and calcification of VSMCs (Sun et al., 2015; Sun et al., 2019; Sommer et al., 2021) and vascular remodeling (Sun et al., 2015). Therefore we explored the effects of salusin-β on proliferation, migration, fibrosis, and calcification of PASMCs in PH *in vivo* and *in vitro*.

In the present study, we found that compared to control rats, there was a significant increase in PAs media wall thickness and a decrease in lumen diameter*,* resulting in stenosis or even complete occlusion of PAs in MCT-PH rats *in vivo*. Severe vascular fibrosis and proportional vascular calcification of small PAs of PH rats existed, which increased the stiffness of PAs and aggravated vascular remodeling in PH rats. Knockdown of salusin-β in PH rats increased lumen diameter, decreased media thickness and media thickness/lumen diameter, and relieved the vascular fibrosis and calcification of PAs, whereas salusin-β overexpression resulted in more severe vascular remodeling. *In vitro*, we also found that compared with PASMCs derived from control rats, the proliferation, migration, fibrosis, and calcification indices of PASMCs derived from MCT-PH rats were elevated. Overexpression of salusin-β not only further promoted them in PH-PASMCs but also elevated them in control-PASMCs. In contrast, knockdown of the salusin-β in PH-PASMCs decreased the proliferation and migration ability, as well as fibrosis and calcification index. These results indicated that salusin-β promotes the proliferation, migration, fibrosis, and calcification of PASMCs, and that endogenic salusin-β is involved in the mechanisms of these manifestations of vascular remodeling in PH.

Although the exact pathophysiology of PH is still unclear, there is increasing evidence that oxidative stress plays a vital role in the proliferation, migration, fibrosis, and remodeling of pulmonary arteries, leading to the development and progression of PH (Crosswhite and Sun, 2010; Xiao et al., 2020; Sekar, 2021). Furthermore, salusin-β has been reported to increase oxidative stress in VSMCs and induce the migration of VSMCs and intimal hyperplasia following vascular injury (Sun et al., 2015). Through stimulating the production of ROS, salusin-β promotes the foam cells formation and monocyte adhesion in atherosclerosis (Sun et al., 2016a). Our previous studies also have demonstrated that salusin-β regulates blood pressure and vascular function in spontaneously hypertensive rats through provoking the NAD(P)H oxidase derived ROS production in arteries (Pan et al., 2021; Sun et al., 2021). However, whether NAD(P)H oxidase-ROS also mediate the effects of salusin-β in pulmonary hypertension remains unclear. In the current study, compared with control, we found that both NAD(P)H oxidase activity and ROS level of PASMCs derived from MCT-PH rats were significantly increased, and overexpression of salusin-β further elevated them, while knockdown of salusin-β decreased them. In addition, overexpression of salusin-β also increased the NAD(P)H oxidase activity and ROS level in PASMCs derived from control rats. These results suggested that the elevated NAD(P)H oxidase activity-derived ROS production by endogenic salusin-β stimulation in PH might contribute to the proliferation, migration, fibrosis, and remodeling of the pulmonary arteries, which we will study in detail in the future.

In addition, we also found that there was the lack of effects of salusin-β knockdown in either the animals or cells experiments of control rats. We speculated that salusin-β might has not a prominent role under physiological conditions, but its overexpression resulting from other factors under pathological conditions contributed in turn to enhance PH characteristics, which we will study in detail in the future.

## Conclusion

Increased salusin-β activity in PAs in PH rats contributes to proliferation, migration, fibrosis, and calcification of PASMCs, and eventually promotes vascular remodeling. Together with its capacity of promoting the imbalance of vascular contraction and relaxation of PAs, salusin-β plays an important role in elevating the pulmonary arterial resistance and pressure and promotes the pathogenesis and development of pulmonary hypertension. The roles of salusin-β is achieved might by stimulating the production of NAD(P)H oxidase-derived ROS. Reducing endogenous salusin-β may provide a new idea and strategy for the prevention and treatment of pulmonary hypertension in the future.

## Data Availability

The raw data supporting the conclusion of this article will be made available by the authors upon reasonable request.
